# Fractional flow reserve or optical coherence tomography guidance to revascularize intermediate coronary stenosis using angioplasty (FORZA) trial: study protocol for a randomized controlled trial

**DOI:** 10.1186/1745-6215-15-140

**Published:** 2014-04-23

**Authors:** Francesco Burzotta, Antonio Maria Leone, Giovanni Luigi De Maria, Giampaolo Niccoli, Valentina Coluccia, Giancarlo Pirozzolo, Silvia Saffioti, Cristina Aurigemma, Carlo Trani, Filippo Crea

**Affiliations:** 1 Department of Cardiovascular Sciences, Catholic University of the Sacred Heart, Largo A. Gemelli 8, 00168 Rome, Italy

**Keywords:** Angiographically intermediate coronary lesions, Fractional flow reserve, Optical coherence tomography

## Abstract

**Background:**

The management of patients with angiographically intermediate coronary lesions is a major clinical issue. Fractional flow reserve provides validated functional insights while optical coherence tomography provides high resolution anatomic imaging. Both techniques may be applied to guide management in case of angiographically intermediate coronary lesions. Moreover, these techniques may be used to optimize the result of percutaneous coronary intervention. We aim to compare the clinical and economic impact of fractional flow reserve versus optical coherence tomography guidance in patients with angiographically intermediate coronary lesions.

**Methods/Design:**

Patients with at least one angiographically intermediate coronary lesion will be randomized (ratio 1:1) to fractional flow reserve or optical coherence tomography guidance. In the fractional flow reserve arm, percutaneous coronary intervention will be performed if fractional flow reserve value is ≤0.80, and will be conducted with the aim of achieving a post-percutaneous coronary intervention fractional flow reserve target value of ≥0.90. In the optical coherence tomography arm, percutaneous coronary intervention will be performed if percentage of area stenosis (AS%) is ≥75% or 50 to 75% with minimal lumen area <2.5 mm^2^, or if a major plaque ulceration is detected. In case of percutaneous coronary intervention, optical coherence tomography will guide the procedure in order to minimize under-expansion, malapposition, and edge dissections.

Cost load and clinical outcome will be prospectively assessed at one and thirteen months. The assessed clinical outcome measures will be: major cardiovascular events and occurrence of significant angina defined as a Seattle Angina Questionnaire score <90 in the angina frequency scale.

**Discussion:**

The FORZA trial will provide useful guidance for the management of patients with coronary artery disease by prospectively assessing the use of two techniques representing the gold standard for functional and anatomical definition of coronary plaques.

**Trial registration:**

Clinicaltrials.gov NCT01824030

## Background

The decision on when and how to treat patients with coronary artery disease represents a major clinical issue and is usually based on a clinical evaluation combined with analysis of coronary angiography. Yet a growing body of evidence suggests that coronary angiography fails to allow detailed assessment of coronary atherosclerotic plaque morphology and severity [1]. As a consequence, in recent years a series of novel technologies have been developed in order to improve the assessment of patients with coronary artery disease [2-4]. Their use may improve the decision making process in patients with equivocal findings at coronary angiography, such as patients with angiographically intermediate coronary lesions (AICL) [1].

The first of these strategies is represented by fractional flow reserve (FFR), defined as the ratio of pressure distal to the stenosis and aortic pressure after induced maximal hyperemia. Its normal value is 1, while a value of 0.75 has been initially purposed as the cutoff in identifying the presence of myocardial ischemia in a validation study of comparison with non-invasive tests, showing a 93% diagnostic accuracy with 100% specificity and an 88% sensitivity [5]. The same cutoff has been confirmed in the DEFER study, in which deferred revascularization of intermediate stenosis was shown to be safe if FFR was above 0.75, with similar long term outcomes in patients in both the percutaneous coronary intervention (PCI) group and those in the defer PCI group [6].

The more conservative cutoff of 0.80 has been tested in two large randomized clinical trials, showing in the drug-eluting stent era, a net clinical and economic advantage from a FFR-guided revascularization strategy [7,8]. On the basis of the FAME [7] and FAME 2 [8] trials, a cutoff value of 0.80 is currently being used in clinical practice in order to identify the ischemic burden associated with AICL, and to guide the choice between revascularization and optimal medical therapy. As shown in Table 1, FFR remains the main technique to be used in clinical studies exploring the impact of adjunctive invasive assessment strategy in guiding the management of patients with AICL.

**Table 1:** Main studies about impact of adjunctive intracoronary techniques to guide PCI in intermediate coronary lesions

**Study**	**Year**	**Patients**	**Technique**	**Lesions**	**Comparison**	**FUP**	**MACE**	**Recurrent Angina**
DEFER [6]	2007	325	FFR	Intermediate Stenosis	FFR guided PCI FFR guided OMT	5 years	↔ in PCI vs OMT (3.3% vs 7.9%, *P* = n.s)	↓ in OMT vs PCI (43.0% vs 33.0%, *P* = 0.02)
Courtis *et al.*[10]	2008	107	FFR	Intermediate Stenosis	FFR guided PCI FFR guided OMT	13 months	↓ in PCI vs OMT (5% vs 23%, *P* = 0.005)	↓ in PCI vs OMT (9.0% vs 41.0%, *P* = 0.002)
Courtis *et al.*[11]	2009	142	FFR	Intermediate Stenosis on LM	FFR guided PCI FFR guided OMT	14 months	↔ in PCI vs OMT (7.0% vs 16.0%, *P* = n.s.)	↓ in PCI vs OMT (20.0% vs 36.0%, *P* = 0.008)
Nam *et al.*[12]	2010	167	FFR vs IVUS	Intermediate Stenosis	FFR guided PCI IVUS guided PCI	1 year	↔ in FFR vs IVUS (3.6% vs 3.2%, *P* = n.s.)	________
Muller *et al.*[13]	2011	730	FFR	Intermediate Stenosis on LAD	FFR guided PCI FFR guided OMT	40 months	↓ in OMT vs PCI (9.7% vs 26.7%, *P* <0.001)	________
Misaka *et al.*[14]	2011	44	FFR	Intermediate Stenosis	FFR guided PCI FFR guided OMT	53 months	↔ in PCI vs OMT (13.3% vs 6.9%, *P* = n.s)	________

FFR, fractional flow reserve; FUP, follow up; IVUS, intravascular ultrasound; LAD, left anterior descending; LM, left main; MACE, major cardiovascular event; MI, myocardial infarction; OMT, optimal medical therapy; PCI, percutaneous coronary intervention.

On the other hand, frequency domain technique optical coherence tomography (FD-OCT), an intracoronary imaging technology warranting quick and precise evaluation of coronary lumen and the vessel wall [4], has entered into clinical practice, providing promising clinical results when applied to guide PCI [9]. On such a basis, most of interventional cardiology centers are (or are going to be) equipped with one or both of the equipment for FFR and FD-OCT. Due to the absence of dedicated trials (Table 1) [6,10-14], the decision of when to use one of these two tools is left to the operator and/or centre preference. Yet, these two techniques have a completely different approach to plaque evaluation, since stenosis-related flow limitation and plaque morphology may influence different aspects of the natural history of ischemic heart disease. Thus, we designed the present prospective clinical trial aimed at comparing the clinical and economic implications associated with the selection of FFR or FD-OCT in the management of patients with AICL.

## Methods/Design

### Study design

The FORZA study is an open label prospective single centre randomized trial comparing the costs and the rate of adverse clinical outcomes in patients randomized to FFR versus FD-OCT guided PCI on AICL.

Consecutive patients with evidence of AICL, defined as a coronary lesion with a visually estimated percentage diameter stenosis ranging from between 30 and 80% [15], will be prospectively enrolled and randomized to FFR guidance or FD-OCT guidance at a ratio of 1:1.

**Figure 1:**
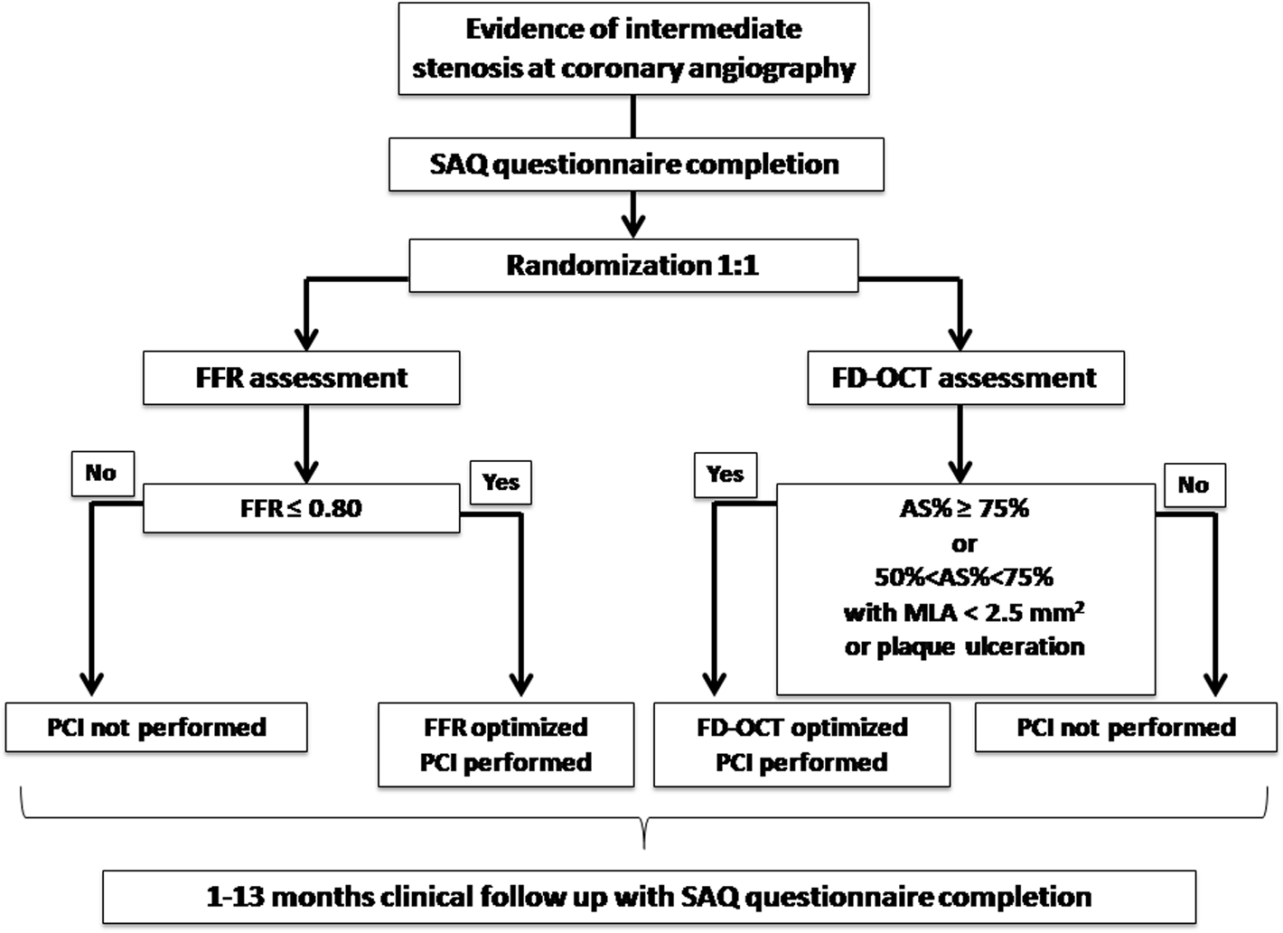
**Study flow chart.** (AS%, percentage of area stenosis; FFR, flow fractional reserve; MLA, minimal lumen area; OCT, optical coherence tomography; PCI, percutaneous coronary intervention; SAQ, Seattle angina questionnaire).

Each patient will complete a Seattle Angina Questionnaire (SAQ) before intervention, at one month, and thirteen months follow up. Details on SAQ are reported in the Methods/Design section. FFR and FD-OCT assessment will be performed at the site of AICL according to technical details reported in the Methods/Design section below. The study flow chart is summarized in Figure 1.

The specific inclusion and exclusion criteria for the study are reported in Table 2.

**Table 2:** Inclusion and exclusion criteria

**Inclusion criteria**	**Exclusion criteria**
**Clinical**	**Clinical**
Age >18 years	Age <18 years
Diagnosis of ischemic heart disease	Inability to give informed consent
	Female with child-bearing potential
**Angiographic**	Life expectancy <12 months
Single vessel disease with angiographically intermediate coronary lesion OR	Factors making clinical follow up difficult (such as no fixed address)
Multivessel disease with only angiographically intermediate coronary lesion OR	Poor cardiac function as defined by left ventricular global ejection fraction ≤30%
Multivessel disease with at least one angiographically intermediate coronary lesion and already treated angiographically critical stenosis	Recent (<7 days) ST-segment elevation Myocardial infarction
	Recent (<48 hours) Non ST-segment elevation myocardial infarction
	Prior ST-segment elevation myocardial Infarction in the territory supplied by the vessel with the intermediate under investigation
	Severe myocardial hypertrophy (interventricular septum thickness >15 mm, ECG Sokolow’s criteria fulfilled)
	Severe valvular heart disease
	Significant platelet count alteration (<100,000 cells/mm3 or >700,000 cells/mm^3^)
	Gastrointestinal bleeding requiring surgery or blood transfusions within the four weeks previous
	History of clotting pathology
	Known hypersensitivity to aspirin, heparin, or contrast dye
	Advanced renal failure with a glomerular filtration rate <30 ml/min
	**Angiographic**
	Multivessel disease with one or more untreated angiographically critical stenosis or coronary occlusion.
	Lesions in coronary artery bypass grafts
	Multivessel disease requiring coronary aortic bypass graft intervention

### Study endpoints

The occurrence of significant residual angina (<90 score at SAQ angina frequency scale) at 13 months represents the primary endpoint of the study.

Major cardiovascular events (MACE), defined as the occurrence of death, myocardial infarction (MI) and target vessel revascularization (TVR), will be prospectively assessed. Since the rate of MACE is anticipated to be low, and most of the procedures in patients with AICL are performed with the aim of improving symptoms, the recurrence of significant angina (defined as an SAQ score <90 on the angina frequency scale) will be prospectively assessed as well.

The first secondary endpoint will be the combined endpoint of recurrence of significant angina or MACE at 13 months follow up. However, in the case of a MACE rate absolute difference of >1% between the two study arms, this combined endpoint will be considered as the primary endpoint of the study.

A further secondary endpoint will be the global costs of the strategy (FFR or FD-OCT guided) at one and thirteen months follow up.

### Procedure description

A guiding catheter (shape and size chosen by the operator) will be placed at the coronary ostium. Then, according to randomization, FFR or FD-OCT assessment will be performed as described below. PCI will be performed at the same time or in a second procedure (‘staged procedure’) according to the decision of the operator only if the pre-specified FFR or FD-OCT criteria are fulfilled, otherwise the patient will be discharged on optimal medical treatment as recommended by international guidelines [16]. The decision regarding pre- and post-dilation, and the type and number of stents needed to cover all of the diseased segment will be left to operator’s discretion. Thereafter, FFR or FD-OCT will be performed and further optimization of PCI procedure will be performed as described below.

### FFR guidance

After intravenous administration of heparin 100 IU/kg, a 0.014 inch pressure monitoring guidewire (Pressure Wire Certus, St. Jude Medical, St. Paul, New Mexico, United States) will be calibrated and advanced into the guiding catheter until the pressure transducer will be just outside its tip, allowing equalization of the pressure measured by the sensor on the guidewire with that measured by the guiding catheter. The guidewire will then be advanced beyond the AICL under examination. Special attention will be paid to avoid arterial pressure wave damping, non-selective cannulation of coronary ostia, and variations in the position of the pressure wire tip. FFR will be calculated as the lowest ratio of distal coronary pressure (Pd) divided by aortic pressure (Pa) after achievement of maximal hyperemia, obtained using intravenous or intracoronary adenosine according to our hospital’s internal protocol [17]. The femoral or brachial vein will be used for intravenous administration of 140 μg Kg-1 min-1 adenosine and maximal hyperemia will be assumed at least after 60 seconds in the presence of stable systemic blood pressure decrease compared to baseline remaining for at least 10 heart-beats. In case of intracoronary infusion, incremental boli of intracoronary adenosine (60, 300 and 600 μg) will be administered with each next dose given at least 60 seconds apart, or after returning to baseline hemodynamic conditions. Each administration will be performed within 5 to 10 seconds and rapidly flushed by saline solution. The following higher dose will not be administered in the case of an atrio-ventricular block lasting more than 5 seconds. In such cases, intravenous adenosine will be used to induce maximal hyperemia [17]. Similarly, in the case of FFR values between 0.81 and 0.83 with 600 μg intracoroary adenosine, FFR will be retested using intravenous adenosine [17]. Finally, an FFR value of ≤0.80 will be considered abnormal. When more than one stenosis is present in the same artery, a pull-back maneuver under maximal hyperemia to determine the exact location and physiological significance of sequential stenosis will be adopted.

If an FFR value of >0.80 is measured on the target AICL, patients will be treated with optimal medical therapy only. If an FFR of ≤0.80 is obtained, patients will undergo a FFR-guided PCI. This means that the patient will undergo conventional PCI with stenting of the AICL in order to achieve a post-stenting FFR ≥0.90. If post-stenting FFR is <0.90 a further post-dilation of the stent will be performed, and if FFR remains at <0.90, a pull-back of the wire to identify another possible pressure drop and/or a subsequent stent implantation at least 5 mm from the stent will be performed according to physician’s preference.

### FD-OCT guidance

FD-OCT images will be acquired at the site of AICL with a commercially available system (C7 System; LightLab Imaging Inc/St Jude Medical, Westford, Massachusetts, United States) after the FD-OCT catheter (C7 Dragonfly; LightLab Imaging Inc/St Jude Medical, Westford, Massachusetts, United States) is advanced to the distal end of the target lesion. The entire length of the region of interest will be scanned using the integrated automated pull-back device at 20 mm/s. During image acquisition, coronary blood flow will be replaced by the continuous flushing of contrast media directly from the guiding catheter at a rate of 4 ml/s with a power injector in order to create a virtually blood-free environment. All images will be recorded digitally, stored, reviewed and analyzed with proprietary software (LightLab Imaging) after confirming proper calibration settings of the Z-offset. Immediately after confirmation of successful FD-OCT image acquisition, the following measures will be performed: minimal lumen area (MLA) (defined as cross section area at the smallest lumen area level), proximal reference lumen area (pRLA) (defined as cross section at the frame with largest lumen within 10 mm proximal to MLA and before any major side-branch), distal reference lumen area (dRLA) (defined as cross section at the frame with largest lumen within 10 mm distal to MLA and before any major side-branch) and mean reference lumen area (mRLA) (defined as (pRLA + dRLA)/2).

On the basis of these parameters, AS% will be calculated using the following formula: (mRLA-MLA)/mRLA × 100.

PCI will be performed when one of the following conditions will be present: AS% ≥ 75%, AS% from 50% to 75% with MLA <2.5 mm^2^, and/or major plaque ulceration evidence at FD-OCT. Major plaque ulceration will be defined as a recess in the plaque beginning at the luminal-intimal border [4]. The described criteria have not been used in the past and have been developed for the present study. At the time the protocol was conceived, only three small studies have directly compared FD-OCT with FFR in order to find a cutoff for one of the anatomic parameters provided by FD-OCT in order to predict FFR [18-20]. Nevertheless a definite cutoff has not been identified and a broadly poor accuracy of FD-OCT in detecting a FFR of ≤0.80 has been described.

For this reason the definition of FD-OCT criteria for this study was based on the concept that no unique cutoff value of any parameter is currently known. In particular, the percentage and small residual cross-section area are known to correlate independently with the functional significance of coronary stenosis [21]. Overall, the accuracy of MLA seems to compare favorably with that of AS%, but MLA cutoffs are known to differ across different coronary vessels and study populations [22].

Moreover, when the AS% is above 75 to 80%, false positives are virtually absent [18], meaning that at these levels AS% is probably a reliable cutoff for intervention (first criterion). When such an extreme situation is not present, we decided to improve the accuracy by combining small MLA with appreciable AS% (second criterion) in order to limit false positives (and indirectly adjusting for vessel size). In dealing with a gray zone for the AS% parameter, a looser MLA cutoff of 2.5 mm^2^ has been chosen in this case in order to reduce the possibilities of false negative.

Finally, since some adverse events may occur in patients with FFR-negative lesions (0.2% occurrence of MI and revascularization rate of 3.2% in FFR >0.80 group of the FAME study, and 3.3% occurrence of combined primary endpoint in the defer group of the DEFER study) [6,7] and untreated lesions with complex morphology may be responsible, we hypothesize that stenosis with the typical features of complicated atherosclerosis (as detectable by FD-OCT) may particularly benefit from treatment (third criterion). In this case only major plaque ulceration was considered as a criterion as it is the hallmark of existing plaque instability. The presence of a thin fibrous cap atheroma (TCFA) was not identified as a criterion for PCI or AICL. Indeed, even if TCFA are associated to possible future plaque instability and may have a prognostic relevance [23], no randomized studies have so far shown a clinical benefit in a preventive stenting strategy for this kind of lesion.

In FD-OCT patients undergoing PCI, FD-OCT will also be used to check PCI results and to guide further optimization of the PCI results, if required. In particular, after the procedure a FD-OCT run will be performed and further intervention (balloon dilation and/or stent implantation) will be performed in the presence of: major stent strut malapposition (defined as a distance between the strut and vessel wall of greater than 350 μm or <350 but >200 μm for a length >600 μm (appreciable in >3 contiguous frames at a pull-back speed of 20 mm/sec) [9,24]), major stent under-expansion (defined as: in-stent minimal cross-section area of <75% of the RLA [9,25]) and major edge dissection (defined as dissection >600 μm (appreciable in >3 contiguous frames at a pull-back speed of 20 mm/sec) [24,26]).

The feasibility of FD-OCT guidance for PCI has been recently reported by several authors [9,27] and it has been shown that it may improve prognosis when compared to the ‘classical’ angiographic guidance [28]. The concept of FD-OCT guidance for PCI is based on the possibility that stent-related complications may influence the long term the occurrence of stent failures [29]. Yet, (minor) stent complications such as edge dissections, plaque prolapse, stent under-expansion, and malapposition are frequently detectable by FD-OCT and may evolve positively in the majority of cases managed conservatively [30]. In a retrospective analysis of our FD-OCT procedures, the above mentioned quantitative cutoffs for treatment of FD-OCT detectable complications were found in one third of cases (unpublished data). Thus, we planned to try to correct by further treatment, only such ‘major’ stent complications among the minor ones detected by FD-OCT.

### Post-procedural management

All patients will undergo cardiac damage markers (Creatin-kinase-MB and Troponin I) assessment before the procedure (when feasible), at 6 and 24 hours after PCI. Thereafter, further blood samples will be performed only if clinically indicated. After PCI, patients will be given aspirin (75 to 100 mg/die) on an ongoing basis and clopidogrel (75 mg/die) according to the European Society of Cardiology Guidelines [16].

### Data set and clinical outcome measures

Clinical data (risk factors, medical history) of enrolled patients will be collected in a dedicated electronic database at the time of the intervention procedure. The date of discharge will be systematically recorded. After discharge, patients will be contacted by phone or ambulatory visit at 1, 6, and 13 months. A follow up coronary angiography will be performed only in the case of typical symptoms or signs of ischemia on noninvasive testing, whereas repeat revascularization will be undertaken in the case of significant (>50%) angiographic stenosis.

In the case of suspected adverse clinical events, pertinent medical records will be carefully reviewed to ascertain the occurrence of MACE. Deaths of an unknown cause will be considered of cardiovascular origin. MI will be defined according to the third universal definition of myocardial infarction by a combination of chest discomfort, typical ECG modifications and the dynamic increasing of troponin T (>99^th^ percentile upper reference limit) related to atherosclerotic plaque rupture, ulceration, fissuring, erosion, or dissection with resulting intraluminal thrombus in one or more of the coronary arteries (Type 1 MI) [31]. Post-procedural MI will be defined as a rise of creatinin-kinase MB more than five times the 99^th^ percentile upper reference limit during the 48 hours after PCI [32]. Thereafter, peri-procedural MI (Type 4a MI) will be defined by a combination of symptoms, typical ECG modifications and elevation of troponin values of more than five times the 99th percentile upper reference limit in patients with a normal baseline, or a rise of troponin values of greater than 20% if the baseline values are elevated, and are stable or falling [31].

TVR will be defined as repeated clinically-driven revascularization by either PCI or coronary-aortic bypass grafting involving the treated vessel. Stent thrombosis will be classified by the Academic Research Consortium definition as definite, probable, or possible and as early (0 to 30 days), late (31 to 360 days), or very late (over 360 days) [33].

Detailed clinical assessment of angina status will take place at one and thirteen months by asking the patients to fulfil the SAQ, a disease-specific functional status measure to quantify the physical and emotional effects of coronary artery disease [34]. It consists of a questionnaire of eleven questions grouped into five main scales measuring clinically important dimensions of coronary artery disease: physical limitation, angina stability, angina frequency, treatment satisfaction, and disease perception. SAQ is scored by assigning each response an ordinal value, beginning with 1 for the response that implies the lowest level of functioning, and summing items within each of the five scales. Scale scores are then transformed to a 0 to 100 range. Because each scale monitors a unique dimension of coronary artery disease, no summary is generated. Yet, due to the evidence of overlaps between the five assessment scales, a <90 score on the angina frequency scale will be used to define ‘significant residual angina’ (see primary study endpoints session) [34].

### Costs analysis

The costs of the procedures will be calculated by determining the amount of guiding catheters, regular wires, pressure wires, FD-OCT-catheters, balloon dilatation catheters, stents, antiplatelet therapy, adenosine, contrast media, and hospital days used for each patient’s procedure. These will be multiplied by the cost of each resource in Euros. Personnel and laboratory time costs will not be included because assumed to be similar between the two strategies.

Costs of repeated PCI without myocardial infarction, repeated PCI in the setting of a non peri-procedural myocardial infarction and coronary artery bypass grafting will be based on the Italian National Health System’s reimbursement rate per diagnosis-related group.

### Regulatory issues

The study protocol was conceived in September 2012, approved by the Ethical Committee of the Catholic University of the Sacred Heart, Polcilinico A. Gemelli Rome, and registered to clinicaltrial.gov (Reference number NCT01824030). The study will be performed in accordance with the ethical standards of the responsible committees on human experimentation and with the Helsinki Declaration. Each patient willing to participate to the study will sign an informed consent module describing aims of the study, features and risks related to the procedure.

### Sample size calculation for clinical outcome

FFR-based treatment has proven to be superior to standard (based on angiography only) treatment by reducing the occurrence of MACE in the long-term [35]. Yet angina relief (an important determinant of quality of life in patients with ischemic heart disease) was not ameliorated by FFR guidance in a recent trial [7]. Thus, the FORZA trial aims to test if FD-OCT guidance may help to improve the clinical management of patients with ischemic heart disease and inconclusive results at coronary angiography. Since hard clinical endpoints are expected to have low frequency and PCI (especially in patients with good prognosis) is performed with the aim of improving the quality of life by eliminating angina and improving heart function, the occurrence of significant residual angina (<90 score at SAQ angina frequency scale) at 13 months has been considered as the primary endpoint.

For sample size calculations, we focused our attention on the clinical outcome at 13 months (a timepoint chosen assuming that most of patients had completed their 12-month double antiplatelet therapy one month before). Then, based on the FAME trial [7], we anticipate 20% of patients will be suffering from persistent angina at follow up in the FFR guidance group. Thus, 20% of patients are expected to have reached the primary endpoint at 13 months in the FFR guidance group. For the present study, we hypothesize that FD-OCT guidance could be able (due to the treatment of a greater number of stenoses and the optimization of stenting procedures which may reduce the rate of untreated angina-causing lesions) to reduce the occurrence of the primary endpoint by 50%. As a consequence, a total number of 400 patients (200 randomized to FFR guidance and 200 to FD-OCT guidance) has been calculated to be needed with an alpha error of 5% and a beta error of 20%.

This sample size is also suitable to satisfy the secondary endpoint of the study, represented by a combination of MACE and/or relief from angina at 13-month follow up. Indeed we assume to have a 5% rate of MACE in the FFR guidance group, in line with the rate observed in a previous study of patients with intermediate lesions treated on the basis of FFR [10]. Thus, combined with the 20% of patients suffering persistent angina at follow up in the FFR guidance group, 25% of patients are expected to have reached the secondary endpoint at 13 months in the FFR guidance group. Since we expect a significant reduction in angina but not in MACE occurrence in the FD-OCT patients, we assume a 50% reduction (exclusively due to angina relief) of the secondary endpoint in this group. As a consequence, a total number of 304 (152 randomized to FFR guidance and 152 to FD-OCT guidance) has been calculated to satisfy the secondary endpoint requirements, with an alpha error of 5% and a beta error of 20%.

Finally, since the sample size is strongly dependent on the rate of events observed in the reference group (and figures in the literature are highly variable), when the first 150 patients complete the 13-month follow up, an interim analysis will be performed and eventually an amendment with a novel sample sizing will be calculated in the case of >30% difference between expected and observed rates.

### Sample size calculation for peri-procedural management costs

The costs for each arm are anticipated to be the combination of diagnostic procedural costs, interventional procedures (eventually derived from the diagnostic ones) and costs related to events occurring during the follow up. On the basis of the NASCI study, we expect a 30% of PCI in the FFR-guided arm [17]. Contrastingly, according to our experience (three years FD-OCT experience at our institution, unpublished data), PCI is anticipated to be performed in about 50% of the FD-OCT-guided arm. Thus considering the mean costs of a FFR-guided PCI (3600€ for PCI + 940€ for the pressure wire + 170€ for adenosine = 4710€) and of an FD-OCT guided PCI (3600€ for PCI + 1600€ for the FD-OCT catheter = 5200€) and the costs of a coronary angiography with FFR (500€ for coronary angiography + 940€ for the pressure wire plus 170€ for adenosine = 1610€) and of a coronary angiography with FD-OCT (500€ for coronary angiography + 1600€ for FD-OCT catheter = 2100€) we expect an overall costs reduction of 30% in the FFR-guidance group compared to the FD-OCT guidance group. Assuming no occurrence, or at least a similar occurrence of MACE, or recurrence of angina at 30 days follow up in both arms, we expect an overall 30% reduction of costs at 30 days follow up in the FFR-guided arm. A total number of 220 patients (110 randomized to FFR guidance and 110 to FD-OCT guidance) with an alpha error of 5% and a beta error of 20% has been calculated to be needed to test this hypothesis.

### Study limitations

The major anticipated limitation of the present study is that it is an open-label and not a double-blinded trial. The treating physicians cannot be blinded since a FFR-guided procedure is fairly different from an FD-OCT-guided PCI.

## Trial status

The trial is in the patient recruitment phase, started on April 2013.

## Abbreviations

AICL: angiographically- intermediate coronary lesions; AS%: percentage of area; dRLA: distal reference lumen area; FD-OCT: frequency domain optical coherence tomography; FFR: fractional flow reserve; MACE: major cardiovascular event; MI: myocardial infarction; MLA: minimal lumen area; mRLA: mean reference lumen area; PCI: percutaneous coronary intervention; pRLA: proximal reference lumen area; SAQ: Seattle angina questionnaire; TVR: target vessel revascularization.

## Competing interests

The authors declare that they have no competing interests.

## Authors’ contributions

FB: conception and design, data collection and analysis, manuscript writing and final approval of the manuscript. AML: conception and design, data collection and analysis, manuscript writing and final approval of the manuscript. GLDM: data collection and analysis, manuscript writing, critical revision and final approval of the manuscript. GN: data collection and analysis, final approval of the manuscript. VC: data collection and analysis, final approval of the manuscript. GP: data collection and analysis, final approval of the manuscript. SS: data collection and analysis, final approval of the manuscript. CA: data collection and analysis, final approval of the manuscript. CT: data collection and analysis, critical revision and final approval of the manuscript. FC: data collection and analysis, critical revision and final approval of the manuscript. All authors have read and approved the manuscript.
